# Early Recognition and Diagnosis of Buried Bumper Syndrome: A Report of Three Cases

**DOI:** 10.1055/s-0039-1692148

**Published:** 2019-08-22

**Authors:** Johan Devia, Juan Jose Santivañez, Mario Rodríguez, Sandra Rojas, Manuel Cadena, Arturo Vergara

**Affiliations:** 1 Fundación Santa Fe de Bogotá, Intensive Care Unit, Universidad del Rosario, Bogotá, Colombia; 2 Fundación Santa Fe de Bogotá, General Surgery, Universidad del Rosario, Bogotá, Colombia; 3 Universidad de Los Andes, Bogotá, Colombia; 4 Fundación Santa Fe de Bogotá, General Surgery, Universidad Surcolombiana, Bogotá, Colombia; 5 Department of General Surgery, Fundación Santa Fe de Bogotá, Bogotá, Colombia

**Keywords:** buried bumper syndrome, percutaneous endoscopic gastrostomy, major complication, internal stump

## Abstract

Buried bumper syndrome (BBS) was described as a complication of percutaneous endoscopic gastrostomy (PEG) that occurs when the internal stump of the probe migrates and is located between the gastric wall and the skin. The increase of compression between the internal stump and the external stump of the gastrostomy tube causes pain and the inability to feed. We present the cases of three patients with BBS managed by the metabolic and nutritional support department. These cases intend to illustrate one of the less frequent complications of PEG, clinical presentation, risk factors, diagnosis, and especially clinical management. Although there are no defined gold standards for its management, the most important points in the management of this condition are early recognition, recommendations to avoid ischemic process at the moment of the insertion of the tube, specific care of the gastrostomy tube, and a periodic nutrition evaluation to avoid overweight, which causes traction and excessive pressure in the gastric wall. It is important for physicians to be aware of the recommendations to prevent BBS and its complications, especially in patients in whom communication can be difficult secondary to their pathologies and comorbidities.

Since the introduction of percutaneous endoscopic gastrostomy (PEG) in the 1980s by Gauderer et al, 1 many advantages were observed of providing adequate nutritional support to patients with limited caloric intake due to the restriction of their underlying disease. 2 However, despite being a safe and effective method, this procedure 3 can present complications that are relatively rare and vary according to their presentation. These are divided into two groups: complications associated with the procedure itself (early), which include aspiration during the procedure, acute hemorrhage, intraperitoneal hematoma, and perforation of the small intestine or colon, and late complications, such as aortogastric fistula, leakage of gastric secretions through PEG, and buried bumper syndrome (BBS). 4 5

We present the cases of three patients with BBS, emphasizing the importance of early diagnostic to avoid possible life-threatening complications. These cases intend to illustrate one of the less frequent complications of PEG, clinical presentation, risk factors, diagnosis, and especially clinical management.

## Case Report: Case 1

A 95-year-old female patient from a geriatric home presented with a history of chronic obstructive pulmonary disease, type 2 diabetes mellitus, hypertension, hypothyroidism, breast adenocarcinoma treated with radical mastectomy and left ganglion emptying, and stroke 5 years ago. Due to her functional status, she required the placement of PEG. She was brought to the emergency room (ER) after having 2 days of burning abdominal pain around the stoma area associated with induration that was exacerbated with the passage of the enteral nutrition. X-ray of the upper digestive tract and total abdominal ultrasound were performed without identifying any specific alterations.

**Fig. 1:**
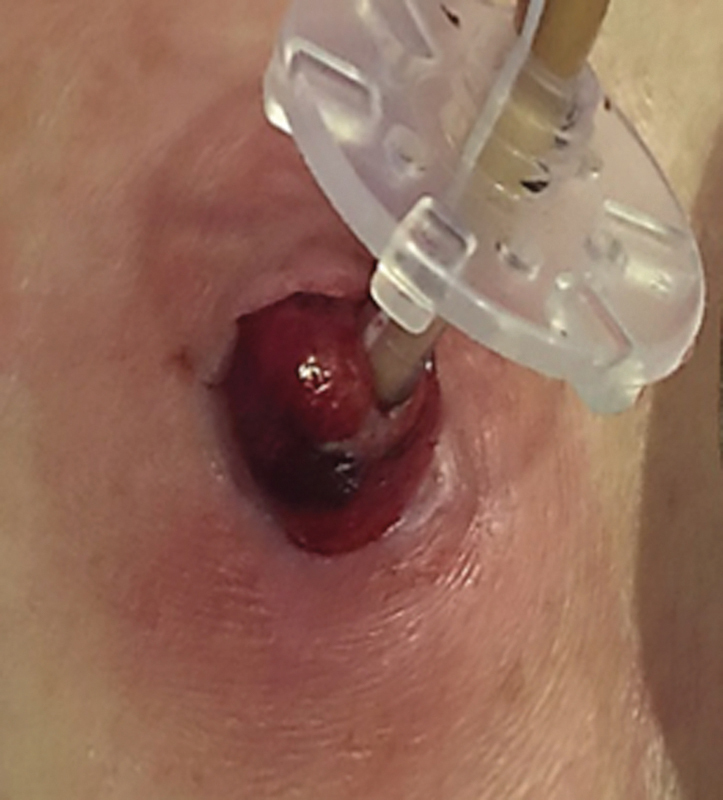
Elevated peristomal edema with serohematic secretion around the catheter gastrostomy embedded in the subcutaneous tissue.

She has persistent abdominal pain, which was, at that point, accompanied by redness with increased temperature around the stoma. Although she was in adequate general conditions with normal vitals, the elevated peristomal edema with serohematic secretion around the catheter gastrostomy was now embedded in the subcutaneous tissue accompanied by intense pain ( Fig. 1 ).

**Fig. 2:**
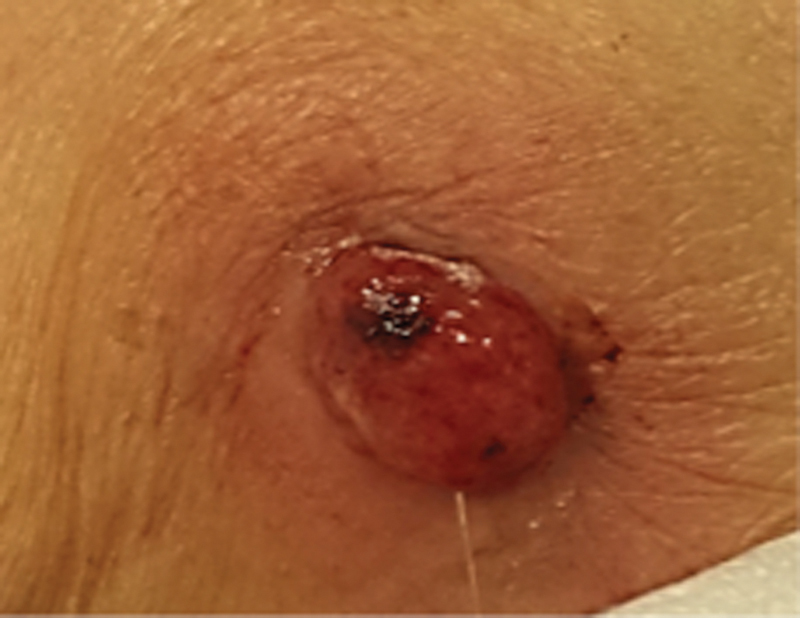
Ostomy with decrease in erythema and without secretion.

Given the gastrostomy mobilization, gastrostomy removal was performed by extracting the buried cannula simultaneously with pulling through of a new system. Antibiotics were in initiated, secretion stains were taken, and parenteral nutrition was ordered. The patient had adequate clinical improvement ( Fig. 2 ). Secretion stains revealed a multisensible *Streptococcus agalactiae* requiring 7 days of intravenous antibiotics. Enteral nutrition was initiated with adequate tolerance. The patient was discharged the next day.

## Case 2

A 97-year-old female patient presented with a history of swallowing disorder secondary to stroke requiring PEG 2 months prior to admission. She was brought to the ER after having partial displacement of the gastrostomy tube, but tube permeability was maintained. No perilesional erythema or leakage was documented. At physical examination, the gastrostomy tube balloon was embedded into the subcutaneous tissue; the diagnosis of BBS was made. The gastrostomy tube balloon was deflated and then reinserted into the gastric cavity without complications. The patient was left under observation along with enteral nutrition reinitiated with adequate tolerance. There were no complications and no signs of inflammatory response, and the patient was discharged.

## Case 3

A 76-year-old male patient transferred from a medium complexity hospital presented with dysphagia for solids and liquids associated with weight loss and grade III malnutrition. He also had a history of benign esophageal stenosis and had a PEG placed from the institution remission. He was treated in the intensive care unit for an unresolved bronchoesophageal fistula that generated episodes of bronchoaspiration and recurrent pneumonias.

**Fig. 3:**
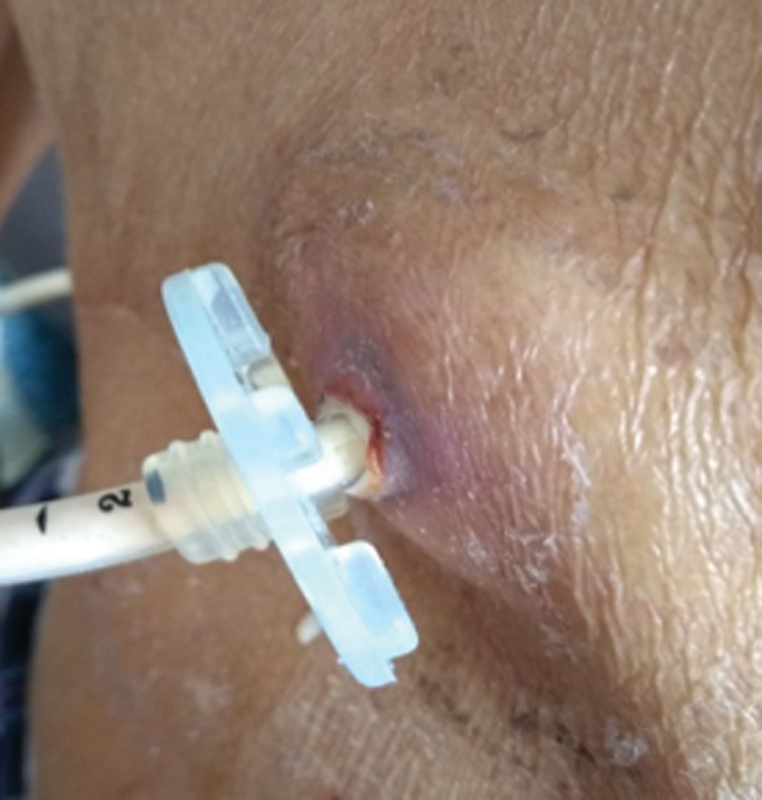
Induration of stoma area associated with leakage through the stoma.

He presented with 2 hours of burning pain in the epigastrium around the stoma area associated with induration that was exacerbated by the passage of the enteral nutrition as well as leakage through the stoma without changes in skin color. Subsequent dysfunction of gastrostomy tube was also observed ( Fig. 3 ). Abdominal pain and gastrostomy tube dysfunction persisted. Physical examination showed an indurated lesion elevated by peristomal edema without secretions, and the gastrostomy tube was embedded into the subcutaneous tissue. Manual maneuvering managed to reinsert the internal gastrostomy stump without complications. Enteral nutrition was reinitiated, with adequate tolerance without new episodes of abdominal pain or leaking.

## Discussion

**Fig. 4:**
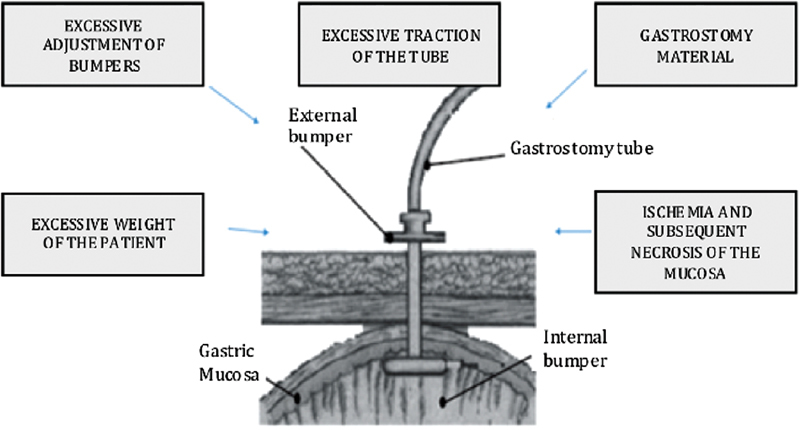
Factors that lead to ischemia and necrosis of the mucosa, facilitating stump migration. 48

BBS was first described in 1988 as a complication of PEG that occurs when the internal stump of the probe migrates and is located between the gastric wall and the skin. The increased compression between the internal stump and the external stump of the gastrostomy tube causes pain and the inability to digest. 5 6 An ischemic process with subsequent necrosis of the gastric mucosa secondary to the excessive pressure exerted by the stump that fix the probe occurs, which weakens the gastric mucosa, and that together with hydrochloric acid and pepsin exacerbates the necrotic process, further weakening the mucosa and allowing migration of the internal stump to be buried between the gastric wall and the skin ( Fig. 4 ). 7

With an incidence of approximately 0.3 to 2.4%, 8 9 10 11 it is considered a major late complication; however, there are documented cases on the appearance of this complication within 3 weeks after performing the procedure. 8 12 In our institution during the last year, 32 percutaneous gastrostomies were performed, of which three cases of BBS were reported, accounting for the 0.96% incidence. This happens because the migration of the internal stump after the PEG has been performed 13 secondary to excessive friction of the probe or because the external stump is fixed too tightly. 14 Its appearance is favored by several risk factors such as: (1) excessive tension between the internal and external bumpers of the gastrostomy tube, (2) increase in hydrochloric acid, which produces physical alterations in the internal bumper, (3) inadequate size or material of the gastrostomy tube, 15 16 (4) inadequate handling and care of the gastrostomy tube, and (5) patient comorbidities such as obesity and chronic cough. 8

The clinical presentation of BBS varies according to the degree of mucosa and gastric wall injury, as well as the immobilization and obstruction of the gastrostomy tube. 17 18 However, more severe presentations of this complication may occur, such as the appearance of fistulas or necrotizing fasciitis, and these are presentations often due to the inability of the patients to manifest symptoms early due to their age or due to their clinical condition. 19 The complications associated with BBS include perforation, peritonitis, 6 20 21 abdominal wall bleeding, 22 23 and abdominal wall abscess, 24 as well as necrosis secondary to pressure and gastric ulcers. 25

Although the majority of these manifestations can be diagnosed clinically based on the history, the physical examination, and the failure to insert and rotate the PEG tube before replacing the external stump, 26 the definitive diagnosis is made by endoscopy since it allows a more accurate localization. 27 However, abdominal ultrasound, 28 endosonography, 29 and abdominal computed tomography (CT) can also be used as diagnostic tools. 30 The findings on endoscopy, which should be performed in all cases in which BBS is suspected, vary according to the duration of symptoms and the time of tube insertion. In early stages, a normal gastric mucosa can be observed with pressure ulcers located under the stump, whereas in more advanced stages, it is common to find an edematous mucosa with an overgrowth of tissue that lines the gastrostomy stump. 31

Orsi et al 32 developed the following classification based on the migration of the internal stump and symptomatology:

- Grade 1: partial migration—asymptomatic presentation or mild symptoms such as abdominal pain or ostomy infection.
- Grade 2: subtotal migration—the patient presents with dysfunction of the tube and extravasation of the nutrition.
- Grade 3: total migration—manifested by tube obstruction.

Although it has been a classification frequently used to serve as a guide for treatment, recently Richter-Schrag et al 33 postulated a new classification based on endoscopic findings:

- IA: extracorporeal or subcutaneous tissue.
- IB: perforated.
- II: partially visible to the mobilization.
- III: totally visible at mobilization, with or without fistula.
- IV: deep, without level of mobilization, with or without fistula.

This classification is much descriptive in terms of the position of the internal stump, although the classification proposed by Orsi et al is more frequently used because it provides simpler information about the location of the internal limit and associated symptomatology, and serves as a guide for treatment.

Regarding the treatment of BBS, despite the contribution provided by the location of the internal bumper, the clinical presentation and comorbidities of the patient also play important roles. Therefore, the management found in the literature has been case reports with individual treatment, 7 in which endoscopic, surgical, and even radiological approaches are described. 34 35 36 However, the management depends on two factors mainly: the type of gastrostomy and the degree of depth to which the internal stump migrates, 37 as this defines the initial approach. If there are no symptoms, the internal stump should always be removed once SBB is diagnosed since failure to do so may lead to the appearance of symptoms and the progression of complications, which make the treatment much more complex. 38 Currently there are no guidelines on which we could base the treatment. We can base our intervention in a conservative approach, the endoscopic therapy, or the surgical therapy. Considering the classification by Orsi et al 32 for treatment purposes, grade 1 has always benefited by an endoscopic approach with external extraction, whereas grades 2 and 3 require a surgical approach.

With the endoscopic approach, if the internal stump is collapsible or malleable, the initial recommendation is to remove the gastrostomy by external extraction 39 ; however, a modification of this technique has been described, in which the gastrostomy tube is cut, through which a guide is passed and travels to the gastric cavity, subsequently, the guide wire is trapped endoscopically and is removed through the oral cavity, where it is attached to a new gastrostomy tube. The portion of the guide wire that is in the abdominal cavity is pulled so that the new gastrostomy tube is inserted into the abdominal wall and, in turn, pushes out the old gastrostomy tube through the abdominal wall. 40 However, this procedure is usually difficult, and in inexperienced hands may result in additional injury. If the internal stump is not collapsible, other techniques for the extraction of the gastrostomy tube have been described, such as the “T technique” by Boyd et al 41 and the “needle-knife” technique by Ma et al 17 for cases in which the internal stump is partially or superficially buried.

When the internal stump cannot be removed by an endoscopic approach, surgical approaches have been described, in which it can be released through a skin incision, 42 or even laparoscopy, when it has migrated to the stomach and an endoscopic extraction is not possible. 43 Likewise, cases have been described in which minimally invasive 44 and radiological techniques are used for their extraction. 45

Although there is no defined gold standard in the management of BBS, the most important aspect of treating this condition is early recognition. Ischemic process should be avoided at the time of tube insertion, specific care should be taken of the gastrostomy tube, and a periodic nutrition evaluation should be made to avoid overweight, which causes traction and excessive pressure on the gastric wall. For the prevention of BBS, it is recommended to leave a space of 1.5 cm between the external stump of the gastrostomy tube and the skin, as well as to externally mobilize and loosen every 2 days. 37 It is also important to provide better care for the gastrostomy system, to constantly follow up these patients, and to encourage effective communication between nutrition specialists, nurses, and the patients and their family. 46 47

## Conclusion

BBS is an unusual complication secondary to PEG; nevertheless, its early recognition is vital for providing the most appropriate approach as well as avoiding its harmful consequences and multiple negative effects on the gastrointestinal tract. Despite the age and multiple comorbidities, the medical history and physical examination were enough to make the diagnosis of BBS. Among the clinical presentations described in the literature, abdominal pain, immobilization of the gastrostomy tube, and inability to pass the enteral nutrition solution were constant in our patients. There is no evidence of leakage or fistulas; however, when leakage or fistulas are present, they occur in advanced cases due to the delay in consultation or in neurological patients and/or advanced age.
